# Supplementary material for: Elasticity of podosome actin networks produces nanonewton protrusive forces
Source: Nat Commun. 2022 Jul 4;13:3842. doi: 10.1038/s41467-022-30652-6 (PMC9253342; doi:10.1038/s41467-022-30652-6)
Supplement: Supplementary file 1 — Supplementary Information [file 41467_2022_30652_MOESM1_ESM.pdf]

Supplementary Information

Elasticity of podosome actin networks produces nanonewton protrusive forces

Marion Jasnin*, Jordan Hervy, Stéphanie Balor, Anaïs Bouissou, Amsha Proag, Raphaël Voituriez, Jonathan Schneider, Thomas Mangeat, Isabelle Maridonneau-Parini, Wolfgang Baumeister, Serge Dmitrieff*, Renaud Poincloux*

* Correspondence to: jasnin@biochem.mpg.de (M.J.), serge.dmitrieff@ijm.fr (S.D.) and renaud.poincloux@ipbs.fr (R.P.)

This PDF file includes:

Supplementary Figures 1-12

Average filament length: $< L >_{[r,\, r+\delta]}$

a

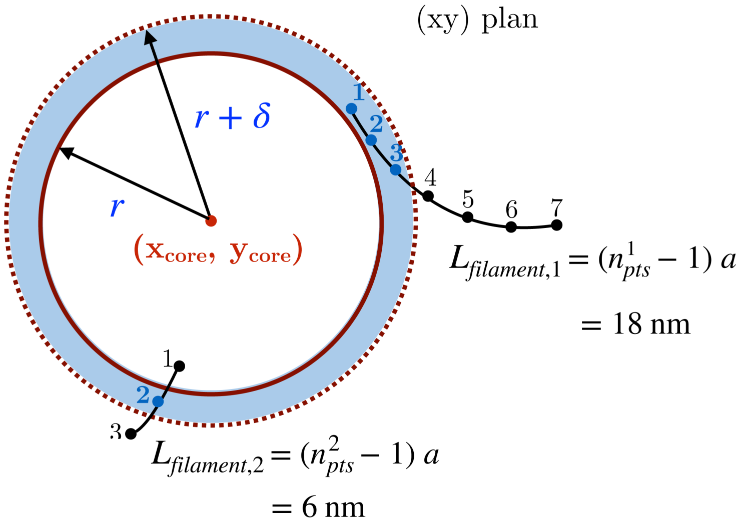

filament 1:

	x	y	z	r
1				
2				
3				
4				
5				
6				
7				

filament 2:

	x	y	z	r
1				
2				
3				

b

$$< L >_{[r,\, r+\delta]} = \frac{1}{n^{tot}_{pts,in}} \sum_{i=1,\, \in [r,\, r+\delta]}^{n_{fil}} n^i_{pts,in}\, L_{filament,i}$$

$$= \frac{1}{4}\left[\underbrace{L_{filament,1} + L_{filament,1} + L_{filament,1}}_{\text{counted 3 times}} + \underbrace{L_{filament,2}}_{\text{counted 1 time}}\right] = 15\ \text{nm}$$

Supplementary Fig. 1 Illustration of the computation of the average filament length as a function of the radial distance. a For each bin [r,r+δ], all filaments with points inside the bin (shown in blue) are identified (left). Each point can be represented both in cartesian and radial coordinates (right). **b** The average length in the bin is the mean of the filament lengths for all filaments with points in the bin, weighted by their number of points in the bin.

Average filament density: $d_{[r,\, r+\delta]}$

a

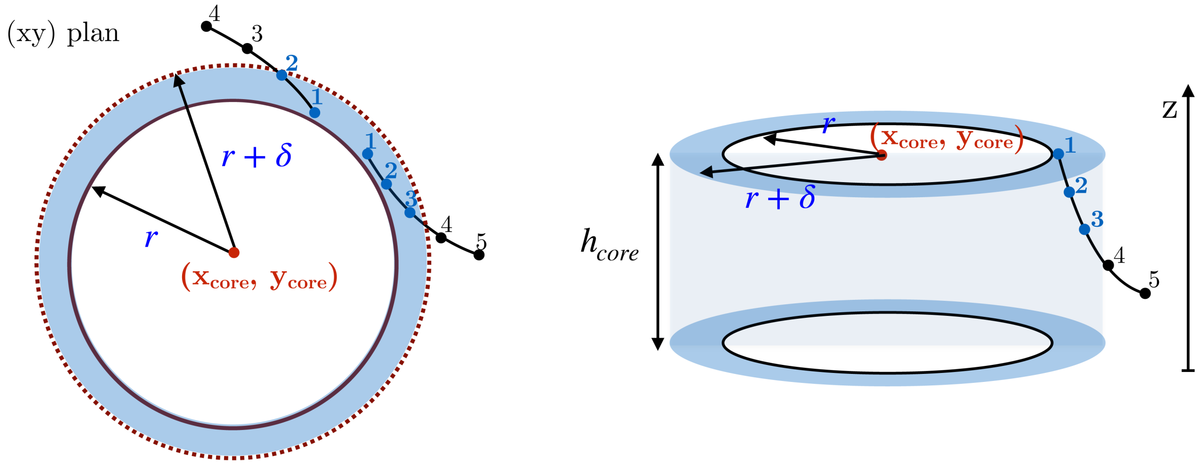

b

$$L_{tot}[r, r+\delta] = \sum_{\substack{i=1, \in [r,\, r+\delta] \\ n^i_{pts,in} > 1}}^{n_{fil}} (n^i_{pts,in} - 1) \times a$$

$$= 3\,\mathrm{nm} + 6\,\mathrm{nm} = 9\,\mathrm{nm}$$

$$d_{[r,\, r+\delta]} = \frac{L_{tot}[r, r+\delta]}{\pi\, h_{core} \left((r+\delta)^2 - r^2 \right)}$$

Supplementary Fig. 2 Illustration of the computation of the average filament density as a function of the radial distance. a For each bin [r,r+δ], the points inside the bins (shown in blue) are identified for each actin filament. **b** The total actin length is calculated in each bin and divided by the bin volume to compute the density.

a

Local filament orientation:

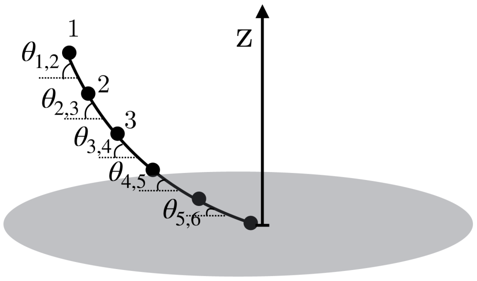

	x	y	z	r		
1						
					$\theta_{1,2}$	$r_{1,2}$
2						
					$\theta_{2,3}$	$r_{2,3}$
3						
					$\theta_{3,4}$	$r_{3,4}$
4						
					$\theta_{4,5}$	$r_{4,5}$
5						
					$\theta_{5,6}$	$r_{5,6}$
6						

$$\theta_{i,i+1} = \frac{180}{\pi} \left| \arcsin \left\{ \frac{z_{i+1} - z_i}{\sqrt{\left((x_{i+1} - x_i)^2 + (y_{i+1} - y_i)^2 + (z_{i+1} - z_i)^2\right)}} \right\} \right|$$

b

Average filament orientation: $< \theta >_{[r,\, r+\delta]}$

$$< \theta >_{[r,\, r+\delta]} = \frac{1}{n^{tot}_{pts,in}} \sum_{i \in [r,\, r+\delta]} \theta_i$$

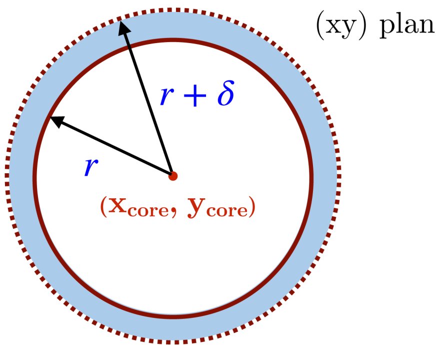

Supplementary Fig. 3 Illustration of the computation of the average filament orientation as a function of the radial distance. a To each segment between two filament points is associated an angle with respect to the membrane plane. **b** The average filament orientation in a bin is the mean of the orientations of all segments inside the bin.

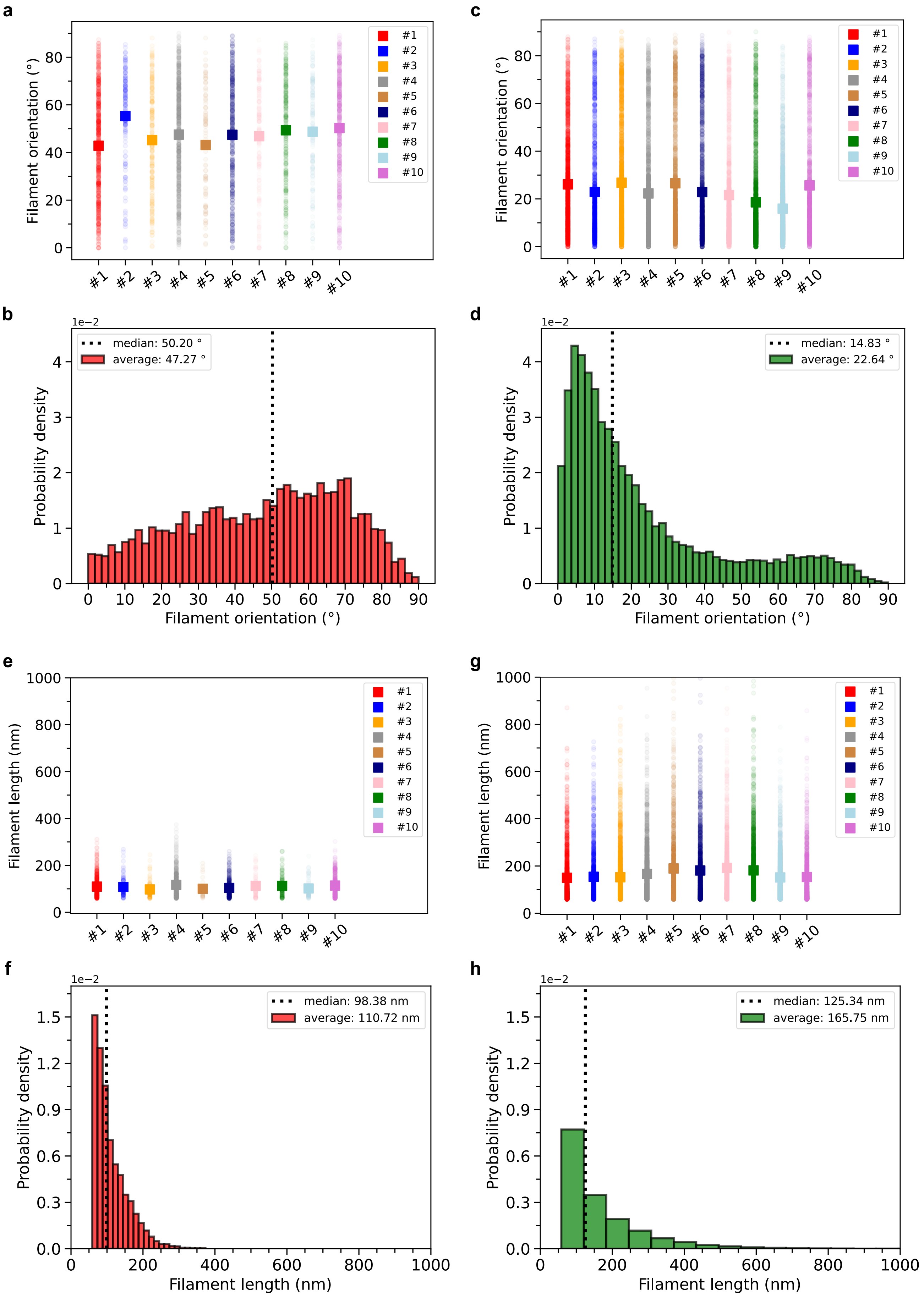
a
Filament orientation (°)
#1 #2 #3 #4 #5 #6 #7 #8 #9 #10
b
1e−2
median: 50.20 °
average: 47.27 °
Probability density
Filament orientation (°)
c
Filament orientation (°)
#1 #2 #3 #4 #5 #6 #7 #8 #9 #10
d
1e−2
median: 14.83 °
average: 22.64 °
Probability density
Filament orientation (°)
e
Filament length (nm)
#1 #2 #3 #4 #5 #6 #7 #8 #9 #10
f
1e−2
median: 98.38 nm
average: 110.72 nm
Probability density
Filament length (nm)
g
Filament length (nm)
#1 #2 #3 #4 #5 #6 #7 #8 #9 #10
h
1e−2
median: 125.34 nm
average: 165.75 nm
Probability density
Filament length (nm)

Supplementary Fig. 4 Orientation and length distributions of the innermost and outermost filaments, respectively, in ten podosomes. a Mean orientation of the innermost filaments, i.e. exclusively within the radial distance range $r \in [r_0-r_s,r_0]$. r_0 and r_s are the inflection point and transition range parameters, respectively, and were obtained from the fit of the orientation as a function of r for each tomogram. Median values are indicated by larger squares. At least 99 inner filaments were measured by tomogram. **b** Corresponding distribution obtained by merging all the data from the top panel into a single dataset. The vertical dashed line indicates the median value of the dataset. **c-d** Same distributions for the outermost filaments, i.e. exclusively within the radial distance range $r \in [r_0+r_s,r_{max}]$ where r_{max} is the highest radial distance between a point filament and the core center. At least 1277 outer filaments were measured by tomogram. **e-h** Same plots as in **a-d** for the filament length. Source data are provided as Source Data files.

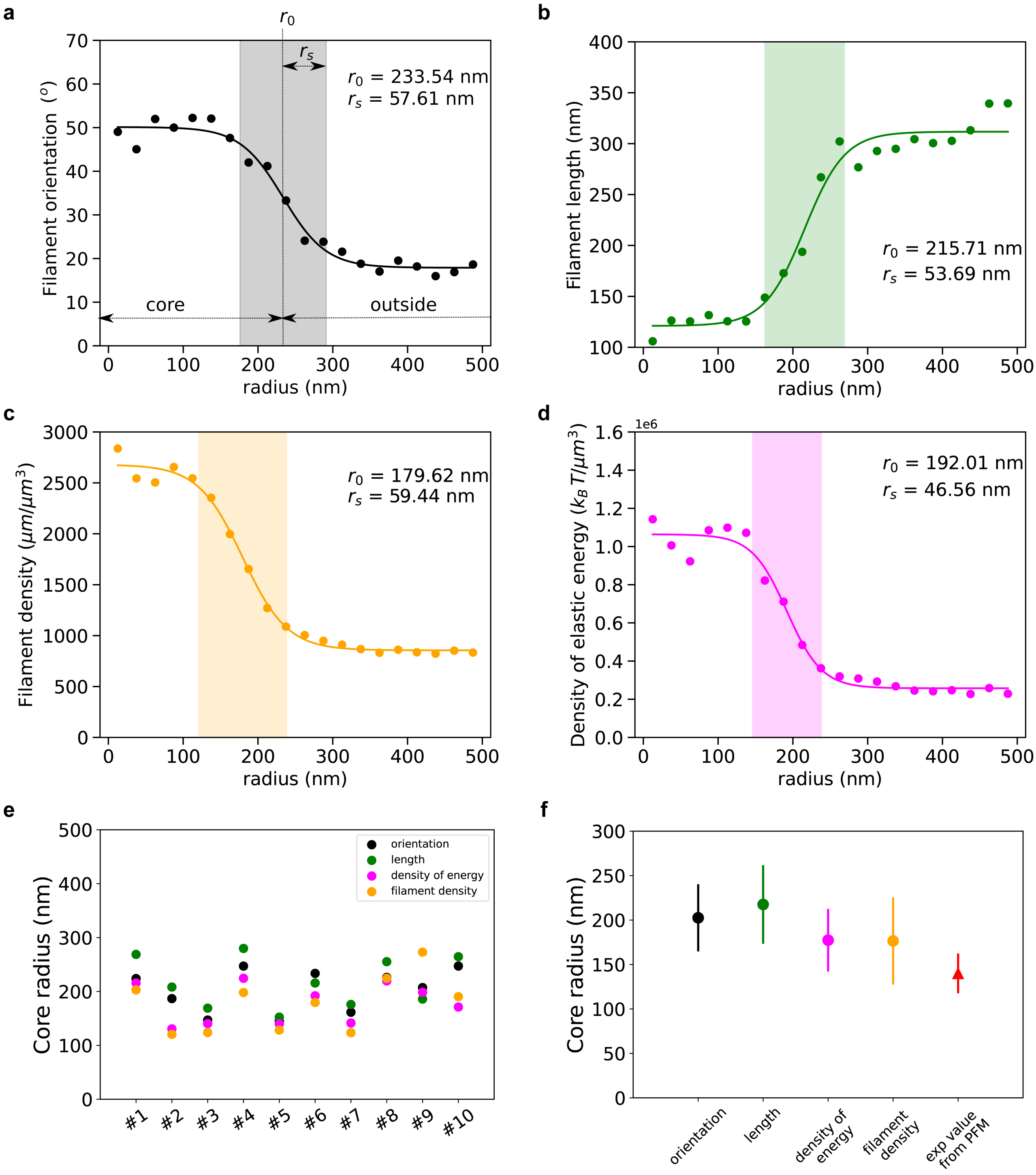

Supplementary Fig. 5 Estimation of the core radius from the distribution of each parameter as a function of the distance to the core center. a-d Filament orientation (**a**), length (**b**), density (**c**) and density of elastic energy (**d**) for podosome #6 with their respective fits are shown. The parameters r_0 and r_s and the transition (colored) zone are indicated. **e** Comparison of the core radius values obtained from the fits of the different parameters as a function of the radial distance for the ten tomograms. **f** Average radius of the podosome core estimated from the quantitative analysis of the cryo-ET data and compared to the reported value from PFM on 30-nm thick Formvar films [17] (mean±SD). Source data are provided as Source Data files.

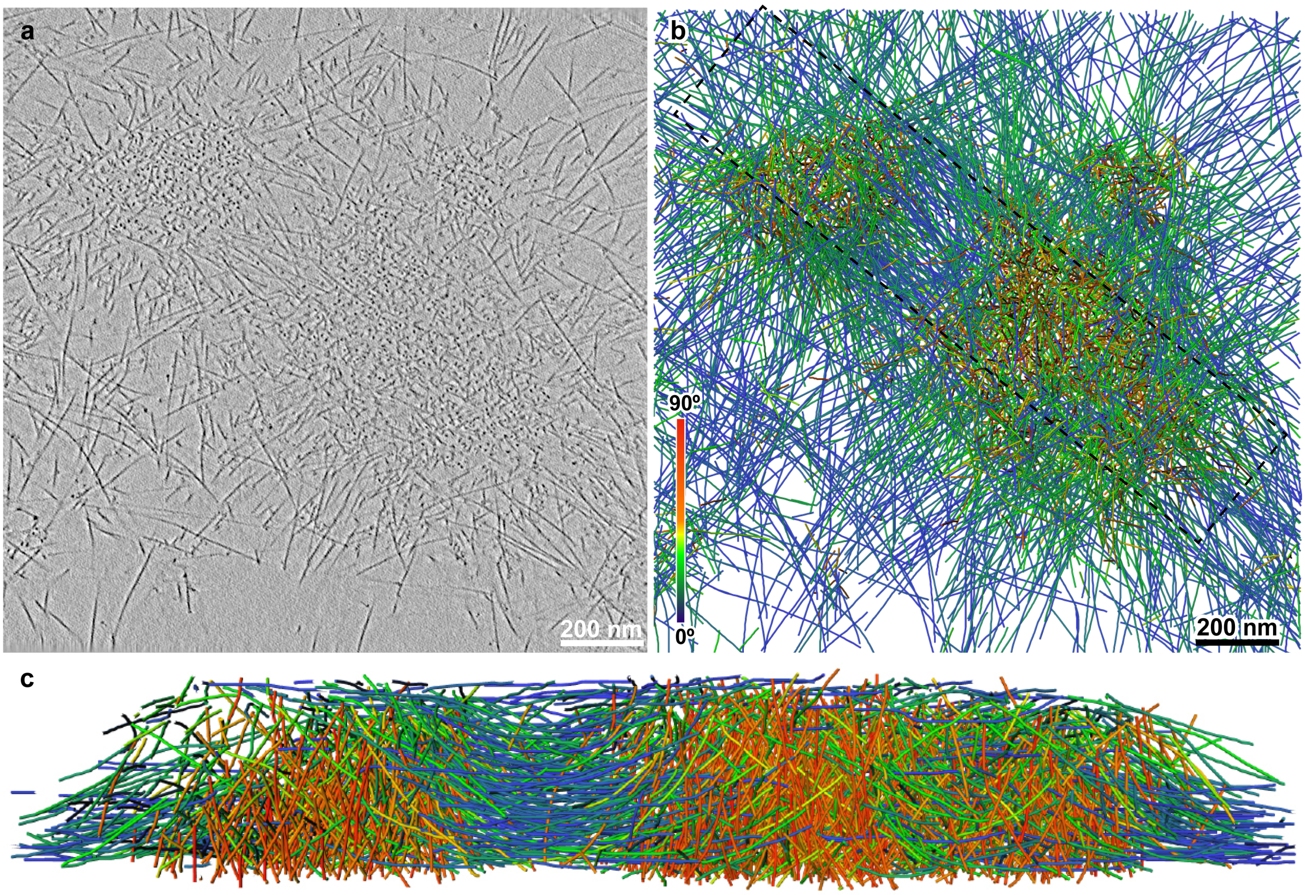

Supplementary Fig. 6 Unroofed podosome architecture revealed by cryo-ET. a Slice from a tomographic volume acquired in a plunge-frozen, unroofed human macrophage revealing neighboring podosomes. The original tomogram has been deposited in the EMDB under accession code EMD-13673. 3 other tomograms showing similar organisations were obtained. **b** Orthographic view of the corresponding 3D segmentation of the actin filaments showing their relative orientation with respect to the basal membrane. **c** Perspective view of the volume indicated by a dotted rectangle in **b** and shown from the left side. The network width is 1.62 µm. See also Supplementary Movie 4.

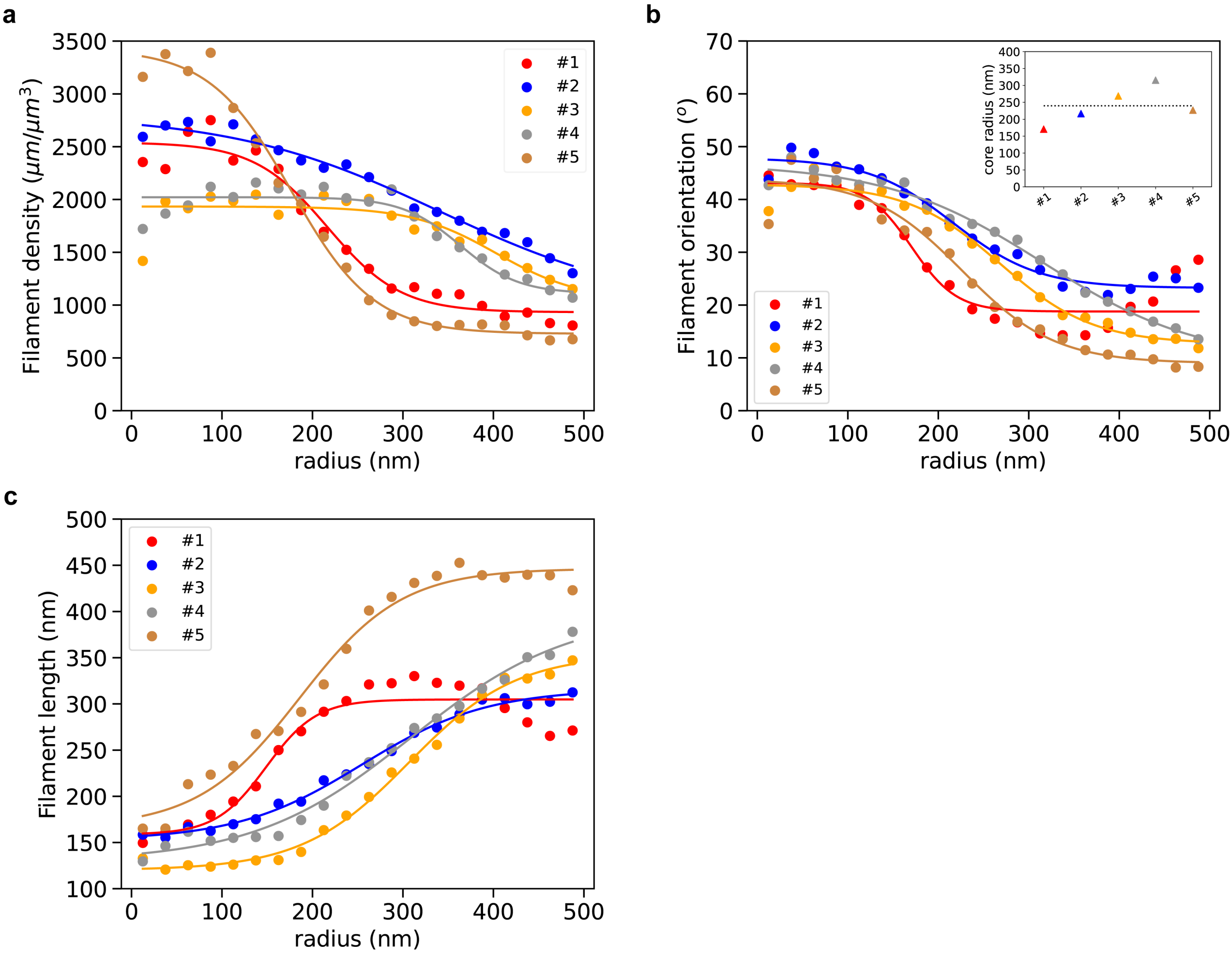

Supplementary Fig. 7 Quantitative analysis of actin filament organization in unroofed podosomes. a-c Filament density (**a**), orientation (**b**) and length (**c**) as a function of the radial distance from the core center for five unroofed podosomes. Inset in **b**: Corresponding values estimated for the core radius (Methods). The podosomes shown in Supplementary Fig. 6 correspond to #1 and #2. Filament density in the unroofed condition is similar to that of the *in situ* data (compare with Fig. 1e), as are filament orientation (41 +/- 21° against 47 +/- 22° in the core, and 23 +/- 21° outside the core, see Fig. 1f) and filament length (107 +/- 50 nm vs 111 +/- 46 nm in the core, and 182 +/- 137 nm vs 166 +/- 120 nm outside, see Fig. 1g). Source data are provided as Source Data files.

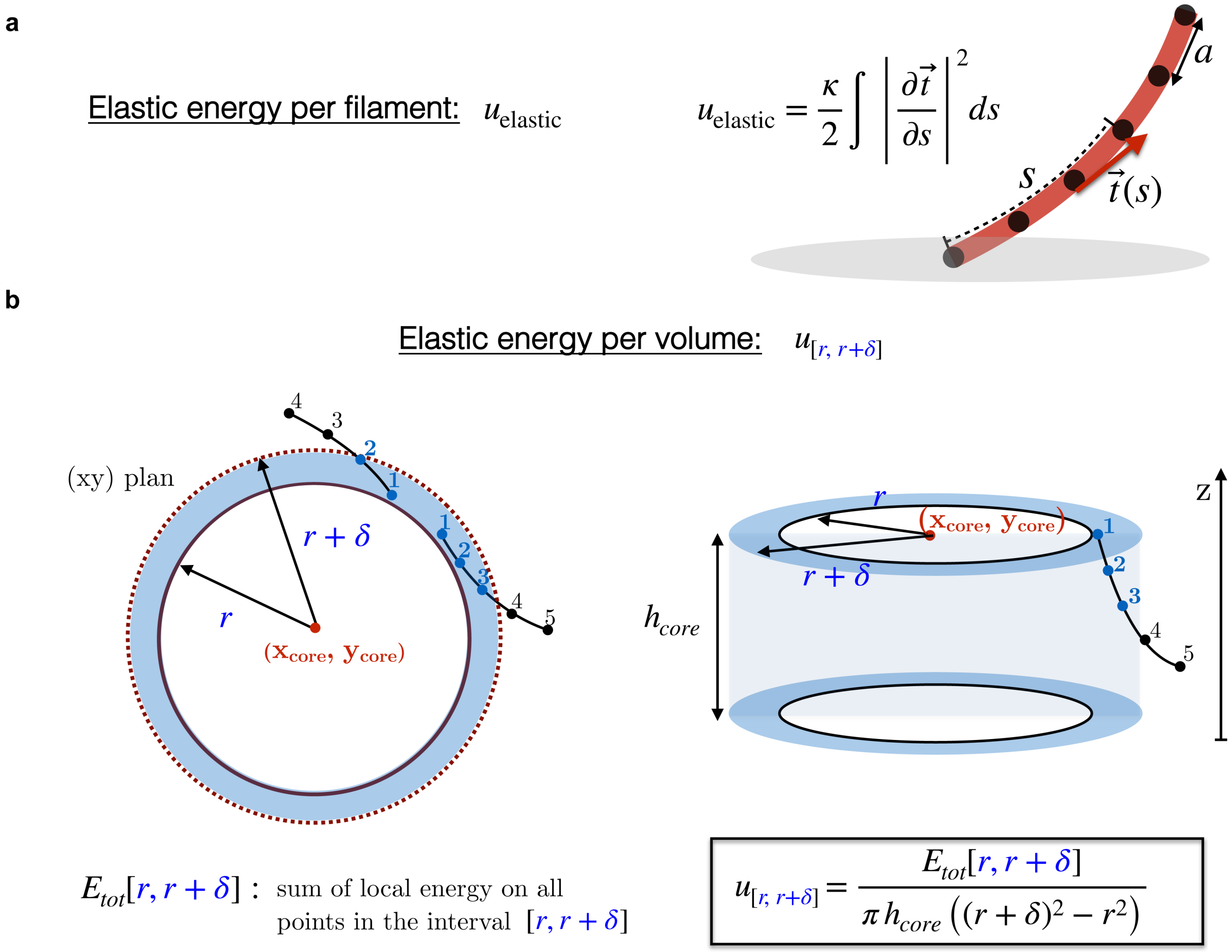

Supplementary Fig. 8 Illustration of the computation of the elastic energy per volume as a function of the radial distance. a Definition of the elastic energy of a filament as the integral of the squared curvature over the filament length. **b** The density of elastic energy as a function of the radial distance is computed as the sum of the elastic energy of all the filament points in the bin divided by the volume.

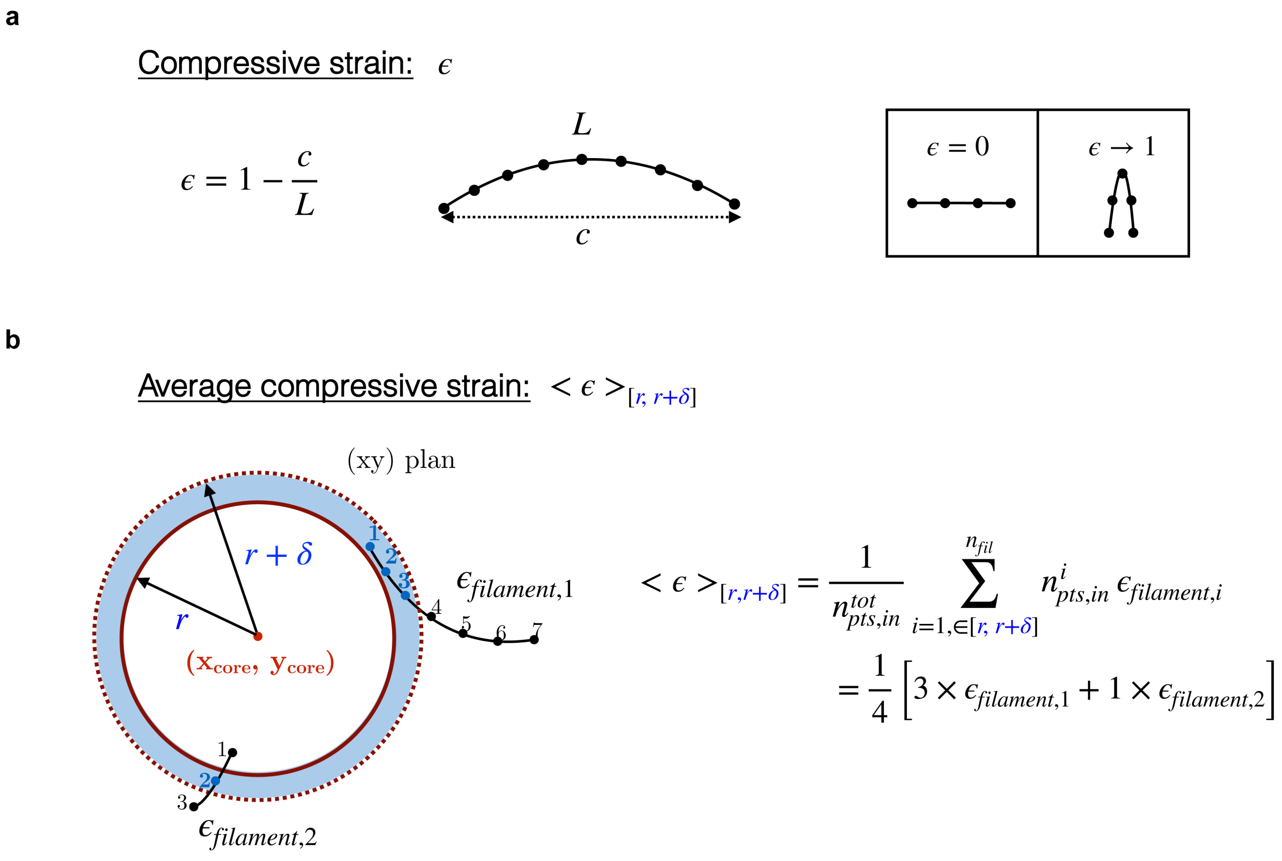

Supplementary Fig. 9 Illustration of the computation of the average compressive strain as a function of the radial distance. a The compressive strain is computed as 1 minus the ratio between the end-to-end distance, *c*, and the filament length, *L*. Thus, a straight filament has a zero strain. **b** The average compressive strain in a bin is computed as the mean of the compressive strains weighted by the number of filament points in the bin.

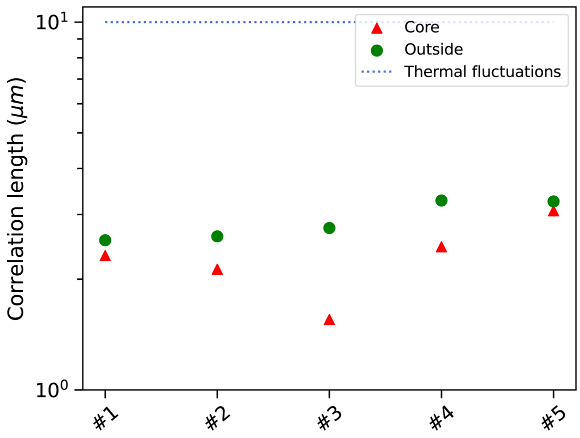

Supplementary Fig. 10 Correlation length estimated in five unroofed podosomes. The correlation lengths inside the core (2.30 μm on average) and outside the core (2.89 μm on average) indicate that filaments are moderately less compressed after unroofing than in their native context (with values of 1.68 and 2.41 μm, respectively, see Fig. 3e). This suggests that the unroofing procedure may have released some of the constraints on the filaments. Source data are provided as a Source Data file.

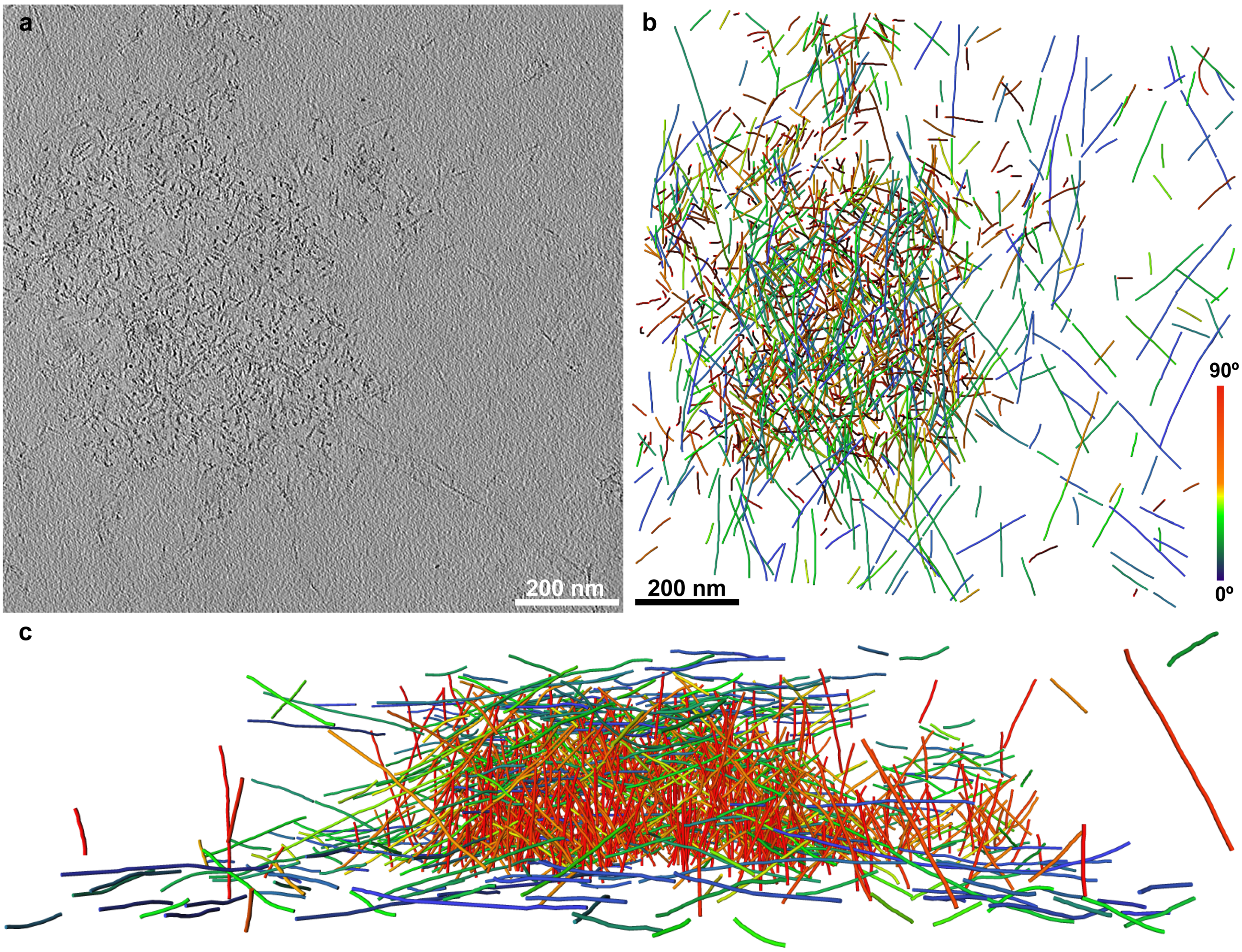

Supplementary Fig. 11 Architecture of unroofed podosomes after cytochalasin D treatment revealed by cryo-ET. a Slice from a tomographic volume acquired in a plunge-frozen, unroofed human macrophage treated with cytochalasin D. The original tomogram has been deposited in the EMDB under accession code EMD-13798. 3 other tomograms showing similar organisations were obtained. **b** Orthographic view of the corresponding 3D segmentation of the actin filaments showing their relative orientation with respect to the basal membrane. **c** Perspective view of the actin filaments from the right side. The network width is 1.26 µm.

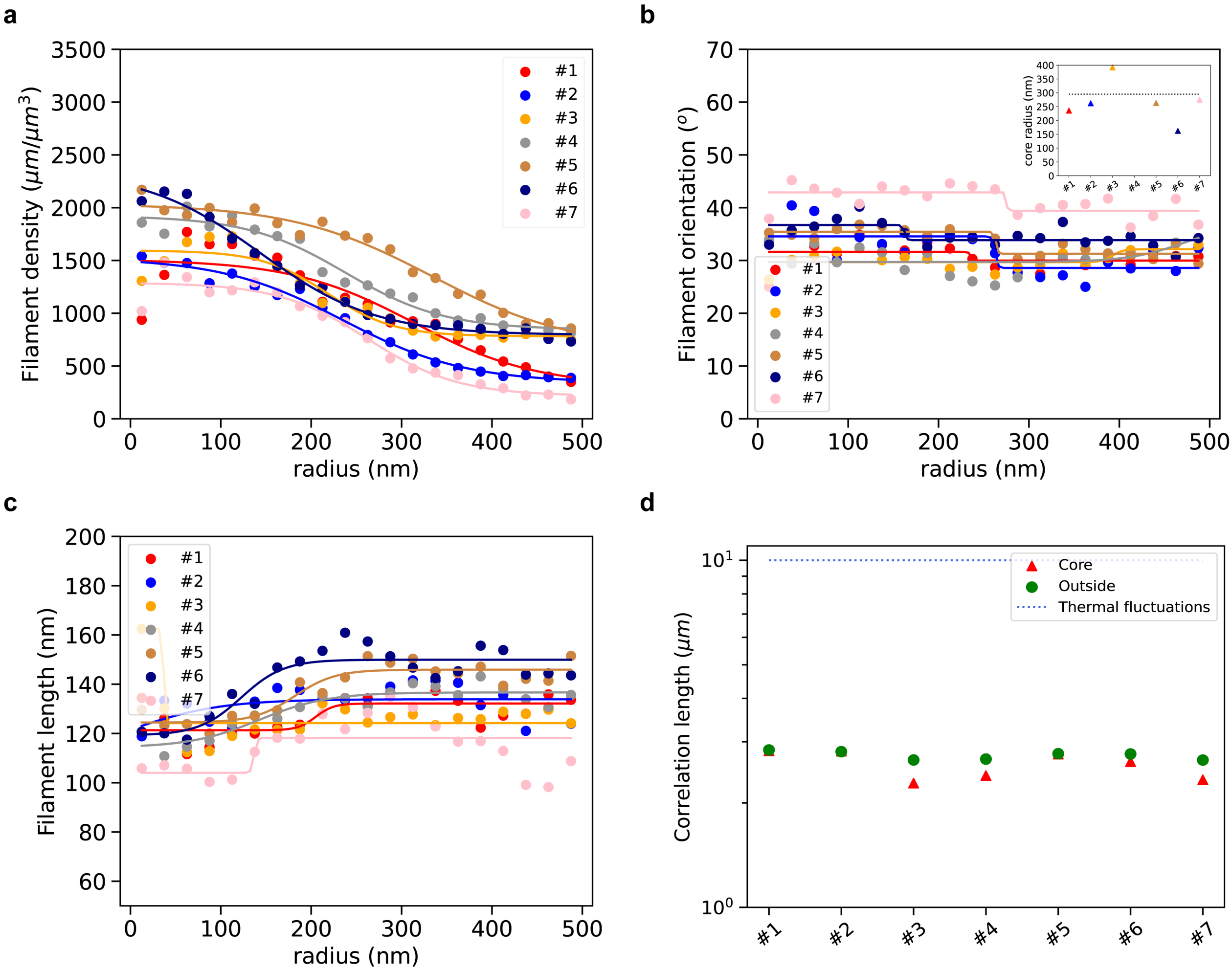

Supplementary Fig. 12 Quantitative analysis of actin filament organization in unroofed podosomes after cytochalasin D treatment. a-c Filament density (**a**), orientation (**b**) and length (**c**) as a function of the radial distance from the core center for seven unroofed podosomes after cytochalasin D treatment. Inset in **b**: Corresponding values estimated for the core radius (Methods). The podosome shown in Supplementary Fig. 11 corresponds to #7. **d** Correlation length in the observed podosomes. The correlation lengths inside and outside the podosome core are similar (2.58 μm versus 2.74 μm on average), suggesting that filaments are no longer under compression. Source data are provided as Source Data files.
