# Supplementary material for: Elasticity of podosome actin networks produces nanonewton protrusive forces
Source: Nat Commun. 2022 Jul 4;13:3842. doi: 10.1038/s41467-022-30652-6 (PMC9253342; doi:10.1038/s41467-022-30652-6)
Supplement: Supplementary file 3 — Description of Additional Supplementary Files [file 41467_2022_30652_MOESM3_ESM.pdf]

File name: Supplementary Movie 1

Description: **F-actin dynamics at podosomes by Random Illumination Microscopy.** Time-series movie by RIM microscopy of a macrophage expressing Lifeact-GFP and Paxillin-GFP, reconstructed at 1-s intervals during 164 s. In macrophages, podosomes have a life span of a few minutes and cover the entire surface of the cell basal membrane, making them easily accessible for cryo-electron tomography.

File name: Supplementary Movie 2

Description: **F-actin and Zyxin dynamics at podosomes by Random Illumination Microscopy.** Time-series movie by RIM microscopy of a macrophage expressing Lifeact-GFP and Zyxin-mCherry, reconstructed at 6-s intervals during 210 s. Zyxin is used to highlight the lateral cables around the podosomes.

File name: Supplementary Movie 3

Description: **Native podosome architecture revealed by *in situ* cryo-ET.** This movie shows the tomogram of the podosome presented in Fig. 1a-d followed by its segmentation. Actin filaments are colored as a function of their orientation with respect to the basal membrane. Intermediate filaments are in brown and membranes in grey. Successive rotation, sectioning and zoom in the segmented volume allow the visualization of the dense organization of the actin filaments in the podosome core.

File name: Supplementary Movie 4

Description: **Architecture of unroofed podosomes by cryo-ET.** This movie shows the tomogram of unroofed podosomes presented in Supplementary Fig. 6 followed by its segmentation. Actin filaments are colored as a function of their orientation with respect to the basal membrane. Successive rotations, sectioning and zoom in the segmented volume reveal the nanoscale actin organization of neighboring podosomes.

File name: Supplementary Movie 5

Description: **Visualization of the actin filaments in the vicinity of the plasma membrane beneath a podosome core.** This movie shows the actin segmentation of the podosome presented in Fig. 2a-b. Core filament segments in the vicinity of the basal membrane are colored as a function of their orientation. A series of rotations, zoomed and unzoomed sections through the segmented volume allow to visualize the number and orientation of the protrusive filaments at the core.

File name: Supplementary Movie 6

Description: **Podosome actin filaments are bent.** This movie shows the actin segmentation of the podosome presented in Fig. 3a. Actin filaments are colored as a function of their mean curvature. Successive zoomed sections through the segmented volume allow to visualize the curvature of the filaments in a native podosome.
