# Supplementary material for: Elasticity of podosome actin networks produces nanonewton protrusive forces
Source: Nat Commun. 2022 Jul 4;13:3842. doi: 10.1038/s41467-022-30652-6 (PMC9253342; doi:10.1038/s41467-022-30652-6)
Supplement: Supplementary file 10 — Reporting Summary [file 41467_2022_30652_MOESM10_ESM.pdf]

nature portfolio

Corresponding author(s): Marion Jasnin, Serge Dmitrieff, Renaud Poincloux

Last updated by author(s): 05/04/2022

Reporting Summary

Nature Portfolio wishes to improve the reproducibility of the work that we publish. This form provides structure for consistency and transparency in reporting. For further information on Nature Portfolio policies, see our Editorial Policies and the Editorial Policy Checklist.

Statistics

For all statistical analyses, confirm that the following items are present in the figure legend, table legend, main text, or Methods section.

n/a	Confirmed	
☐	☒	The exact sample size (*n*) for each experimental group/condition, given as a discrete number and unit of measurement
☐	☒	A statement on whether measurements were taken from distinct samples or whether the same sample was measured repeatedly
☒	☐	The statistical test(s) used AND whether they are one- or two-sided<br>*Only common tests should be described solely by name; describe more complex techniques in the Methods section.*
☒	☐	A description of all covariates tested
☒	☐	A description of any assumptions or corrections, such as tests of normality and adjustment for multiple comparisons
☒	☐	A full description of the statistical parameters including central tendency (e.g. means) or other basic estimates (e.g. regression coefficient) AND variation (e.g. standard deviation) or associated estimates of uncertainty (e.g. confidence intervals)
☒	☐	For null hypothesis testing, the test statistic (e.g. *F*, *t*, *r*) with confidence intervals, effect sizes, degrees of freedom and *P* value noted<br>*Give P values as exact values whenever suitable.*
☒	☐	For Bayesian analysis, information on the choice of priors and Markov chain Monte Carlo settings
☒	☐	For hierarchical and complex designs, identification of the appropriate level for tests and full reporting of outcomes
☒	☐	Estimates of effect sizes (e.g. Cohen's *d*, Pearson's *r*), indicating how they were calculated

Our web collection on statistics for biologists contains articles on many of the points above.

Software and code

Policy information about availability of computer code

Data collection	SerialEM 3.7.0
Data analysis	ImageJ 1.52p<br>K2Align (https://github.com/dtegunov/k2align)<br>TOMOMAN 08042020 (https://github.com/williamnwan/TOMOMAN)<br>MotionCor2 1.2.1<br>IMOD 4.10.18<br>Amira 2019.2 with XTracing extension<br>MATLAB R2015b<br>Podosome analyzer (https://gitlab.com/SergeDmi/podosome-demo)<br>AlgoRIM V1_2 (https://github.com/teamRIM/tutoRIM)

For manuscripts utilizing custom algorithms or software that are central to the research but not yet described in published literature, software must be made available to editors and reviewers. We strongly encourage code deposition in a community repository (e.g. GitHub). See the Nature Portfolio guidelines for submitting code & software for further information.

Data

Policy information about availability of data

All manuscripts must include a data availability statement. This statement should provide the following information, where applicable:

- Accession codes, unique identifiers, or web links for publicly available datasets
- A description of any restrictions on data availability
- For clinical datasets or third party data, please ensure that the statement adheres to our policy

Data supporting the finding of this manuscript are available from the corresponding authors. Five representative tomograms have been deposited in the EMDB under accession codes EMD-13666, EMD-13669, EMD-13671, EMD-13673 and EMD-13798.

Field-specific reporting

Please select the one below that is the best fit for your research. If you are not sure, read the appropriate sections before making your selection.

☒ Life sciences ☐ Behavioural & social sciences ☐ Ecological, evolutionary & environmental sciences

For a reference copy of the document with all sections, see nature.com/documents/nr-reporting-summary-flat.pdf

Life sciences study design

All studies must disclose on these points even when the disclosure is negative.

Sample size	The sample size (10 in situ tomograms from 5 cells; 5 unroofed tomograms from 2 cells; 7 unroofed tomograms from 1 cell treated with cytoD) was chosen because of the practical limitations of the methodology used. It was sufficient to interpret the data in a robust manner according to previous publications (see for example Table S2 in Watanabe et al. Cell 2020 or Supplementary Table 1 in Kiesel et al. Nat Struct Mol Biol 2020).
Data exclusions	Tomograms with insufficent signal-to-noise ratio or insufficient podosome thickness after FIB milling were excluded from the analysis.
Replication	In situ EM data are from 3 isolations from 3 donors, each donor corresponding to an independent experiment, with a total of 5 cells investigated.<br>Control unroofed EM data are from 2 isolations from 2 donors, each donor corresponding to an independent experiment, with a total of 2 cells investigated. Cytochalasin D unroofed EM data are from 1 donor, with 1 cell investigated.<br>All attempts at replication were successful.
Randomization	Donors were randomly selected for isolation of the blood monocytes. Cells were randomly selected for FIB/EM experiments.
Blinding	Blinding was not relevant for this study because it is not a comparative study.

Reporting for specific materials, systems and methods

We require information from authors about some types of materials, experimental systems and methods used in many studies. Here, indicate whether each material, system or method listed is relevant to your study. If you are not sure if a list item applies to your research, read the appropriate section before selecting a response.

Materials & experimental systems

n/a	Involved in the study	
☒	☐	Antibodies
☒	☐	Eukaryotic cell lines
☒	☐	Palaeontology and archaeology
☒	☐	Animals and other organisms
☒	☐	Human research participants
☒	☐	Clinical data
☒	☐	Dual use research of concern

Methods

n/a	Involved in the study	
☒	☐	ChIP-seq
☒	☐	Flow cytometry
☒	☐	MRI-based neuroimaging
